# Metagenome-scale modeling to assess microbiome metabolic complementarity for precision microbiota transplantation therapies

**DOI:** 10.1080/19490976.2026.2725403

**Published:** 2026-09-02

**Authors:** Zetian Zhang, Mark Holton, Daniel M. Ferrer, Arielle D. Tripp, Alexander Richter, Purushottam D. Dixit, Guillaume Urtecho

**Affiliations:** a School of Biomedical Sciences, The University of Hong Kong, Hong Kong SAR, China; b Department of Medicine, Division of Gastroenterology, University of California, San Diego, CA, USA; c Biomedical Sciences Graduate Program, University of California, San Diego, CA, USA; d Department of Epidemiology & Biostatistics, University of California San Francisco, San Francisco, CA, USA; e Institute of Data Science & Biotechnology, Gladstone Institutes, San Francisco, CA, USA; f Department of Biomedical Engineering, Yale University, New Haven, CT, USA; g Systems Biology Institute, Yale University, New Haven, CT, USA

**Keywords:** Fecal microbiota transplantation, community-scale metabolic modeling, bacterial therapeutics, IBS, FMT, irritable bowel syndrome, MICOM, colonization resistance

## Abstract

Fecal microbiota transplantation (FMT) holds therapeutic promise beyond recurrent *Clostridioides difficile* infection, but clinical outcomes remain unpredictable and donor-selection strategies remain limited, in part because the role of donor‒recipient metabolic interactions in shaping the post-FMT community remains poorly understood. Here, we leverage metagenome-scale metabolic modeling to quantify metabolic niche complementarity between donor and recipient microbiomes and predict post-FMT community composition. Using MICOM-derived metabolic models, we show that donor genomes whose metabolic flux profiles are more dissimilar from the recipient community colonize at significantly higher rates in a murine FMT model. In a human IBS trial, the same metric predicted post-FMT community composition via leave-one-out cross-validation and captured known disease-associated alterations in short-chain fatty acid, sulfur, and gas metabolism. We then performed 2,548 *in silico* FMT simulations between IBS-D/M patients and donors from the OpenBiome biobank to evaluate personalized donor screening, identifying super-donors characterized by high taxonomic diversity, broad metabolic niche coverage, and community interaction networks dominated by cross-feeding rather than competition. Together, these results support metabolic niche complementarity as a potential determinant of post-FMT community composition and provide a mechanistic basis for evaluating donor–recipient metabolic compatibility. This framework offers a scalable approach for generating testable hypotheses for personalized donor selection.

## Introduction

The human gut microbiome plays a central role in host health, and disruptions in this complex community, termed “dysbiosis”, have been implicated in numerous diseases.^1^ Fecal microbiota transplantation (FMT), in which microbial stool communities from a healthy donor are transferred to a patient, has emerged as a powerful method to restore microbial balance. This approach has been most successful in recurrent Clostridioides difficile infection (CDI), where FMT achieves cure rates of 80%–90%, and the first standardized FMT products have recently gained FDA approval for the prevention of recurrence.^2^ Beyond CDI, FMT has been explored as a remedy for metabolic and inflammatory conditions (e.g., obesity, diabetes, and IBS), with some reports of symptom improvements.^3^ ^,^ ^4^ However, these broader applications remain experimental, and FMT is not yet routinely used outside of CDI owing to variable patient responses and incompletely understood relationships among donor colonization, microbiome compositional change, and clinical benefits.^5^

Despite FMT’s promise, outcomes differ markedly between recipients. Many patients fail to establish or sustain engraftment of donor bacteria, in part owing to colonization resistance, where the resident microbiota outcompetes incoming strains for nutrient niches.^6^ This niche competition between microbes is thought to be an important determinant of donor microbial colonization. Because colonization success depends on the specific pairing of donor and recipient communities, outcomes should in principle be predictable from features of both communities measured before transplantation, although the exact relationship between colonization and clinical response remains unresolved.^5^ Accordingly, several groups have attempted to predict FMT microbial colonization a priori using microbiome sequencing data and machine-learning models.^7-10^ For example, Smillie et al. modeled engraftment at the level of individual species and strains, showing that whether a given taxon engrafts can be predicted largely from its donor and recipient abundances and how closely related it is to other bacteria in the community.^7^ Large-scale strain-resolved analyses across FMT trials have established that donor strain engraftment depends on recipient, donor, and species-level factors, including pre-FMT recipient dysbiosis, donor and recipient *α*-diversity, and species abundance and oxygen tolerance.^11^ Other models have taken more holistic perspectives focused on donor-recipient complementarity at community and strain-population levels, and concluded that overall dissimilarity between donor and recipient microbiomes is an important driver of FMT colonization patterns rather than properties of any single taxon.^10^ ^,^ ^12^ This concept is based on niche availability and assumes that taxa from more dissimilar communities are more likely to exploit underutilized niches in the recipient. However, given substantial strain-level variation, taxonomic identity alone is insufficient to assign microbes to their metabolic niches. As a result, we still lack practical, mechanistically grounded methods to measure functional compatibility between donor microbes and recipient niches and to use this information prospectively for donor selection.

Ecologically, the gut can be viewed as a mosaic of distinct niches defined by their biochemical resources, spatial locales, and host factors.^13^ ^,^ ^14^ Diverse native microbiota occupy most of these niches, thereby resisting invasion by foreign microbes through competition for nutrients and space.^6^ Thus, new microbes may be more likely to become established if they are able to exploit a niche that is underutilized by the resident community.^15^ Expanding a microbe’s niche range to target resources not consumed by the host’s microbiome has indeed been shown to enable its stable long-term colonization.^16^ While identifying niches within microbiomes is challenging, multi-omics approaches now provide direct insight into such niche dynamics. For example, integrated metatranscriptomic and metatranslatomic analyses have classified gut bacteria into functional guilds and identified their preferred nutrient substrates (niches), enabling the selective manipulation of community members.^17^ While powerful, these empirical approaches are costly, labor-intensive, and cannot easily capture the full metabolic complexity of natural gut communities. There is a pressing need for practical, scalable methods to profile a patient’s microbiome niche structure so that FMT donors can be rationally chosen to fill vacant metabolic niches.

Genome-scale metabolic models (GEMs) offer a promising computational avenue to characterize microbial niches *in silico*. GEMs leverage genomic annotations and biochemical reaction networks to simulate an organism’s metabolic capabilities, thereby predicting which nutrients it can consume and what metabolites it produces.^18^ Recent advances have enabled these models to be scaled up from single strains to whole communities, allowing prediction of microbial activity in complex environments such as the gut.^19^ ^,^ ^20^ For instance, the MICOM framework can integrate dozens of gut bacterial GEMs to simulate community metabolism and growth under defined dietary conditions.^19^ Such community metabolic modeling has already shown that certain gut bacteria maintain conserved “niche structures” across individuals, and it can predict community-level outputs (e.g., short-chain fatty acid production) in different hosts.^21^ Moreover, metabolic modeling has been applied to ecological niche theory, where, by treating a microbe’s simulated metabolic phenotype as a proxy for its niche, one can quantify niche overlap or complementarity between species.^18^ This approach enables direct comparisons of metabolic niches and provides a quantitative way to partition niches among community members. Recently, these models have been used to predict pathogen niches and assess the susceptibility of populations to *Clostridioides difficile.* ^22^

In this work, we leverage metagenome-scale community metabolic modeling to develop a metric, metabolic distance, that quantifies niche complementarity between FMT recipients and donor microbes. We show that metabolic distance captures ecological interactions associated with donor microbial colonization, accurately predicts post-FMT microbiome composition, and identifies predicted metabolic features associated with clinical response. We first validated this framework in a controlled murine FMT system without antibiotic pretreatment, demonstrating that donor microbes with metabolic capabilities orthogonal to the recipient’s microbiome achieve higher colonization success. We then evaluated whether these ecological principles translate to a human clinical trial of FMT for irritable bowel syndrome, where no antibiotics were used, showing that metabolic distance accurately predicts post-FMT microbiome composition. Finally, we extend this framework into an *in silico* donor-selection pipeline that performs 2,548 donor-recipient FMT simulations and identifies mechanistic features associated with super-donors. Together, these findings demonstrate that a minimal set of metabolic parameters, measured before transplantation, can accurately predict post-FMT microbiome composition and associated metabolic phenotypes. By integrating metabolic niche profiling with machine learning, this study lays the groundwork for a new approach to matching FMT donors to patients based on complementary microbiome metabolisms, with the goal of maximizing engraftment and therapeutic outcomes.

## Results

### Metabolic distance robustly predicts engraftment in a murine FMT model

We first asked whether genome-scale metabolic models (GEMs) could capture quantitative differences in metabolic niche use that explain which taxa are engrafted during transplantation. GEMs leverage genomic data to model metabolic phenotypes, predicting each microbe's functional capabilities and ecological role based on simulated nutrient uptake and metabolic outputs under steady-state conditions. We hypothesized that if microbiomes occupy distinct regions of metabolic niche space, then the degree of functional dissimilarity between donor and recipient taxa should predict colonization success, with donor microbes that exploit underutilized niches in the recipient community having a competitive advantage during colonization.

We tested this hypothesis using a previously published murine FMT dataset.^23^ In this work, FMT was performed between all possible pairs of C57BL/6 mice from four different vendors (Jackson Laboratories, Taconic, Envigo, and Charles River), which carried distinct microbiomes, and metagenomic sequencing was used to assess the composition of these communities before and after FMT. Reanalysis reconfirmed substantial variation in engraftment across donor‒recipient pairings. To quantify donor microbial colonization following FMT, we identified donor-enriched metagenome-assembled genomes (MAGs) that were absent or rare before and significantly increased in abundance after transplantation using differential abundance analysis (see Methods). Hence, we refer to MAGs satisfying these criteria as those successfully engrafted throughout this study. We observed that the Envigo microbiota transferred the greatest number of MAGs across all recipients while Taconic communities transferred the fewest MAGs (Figure S1). We reconstructed GEMs from MAGs using CarveMe^24^ and simulated steady-state metabolic fluxes under a high-fiber diet using MICOM.^25^

**Figure 1.**
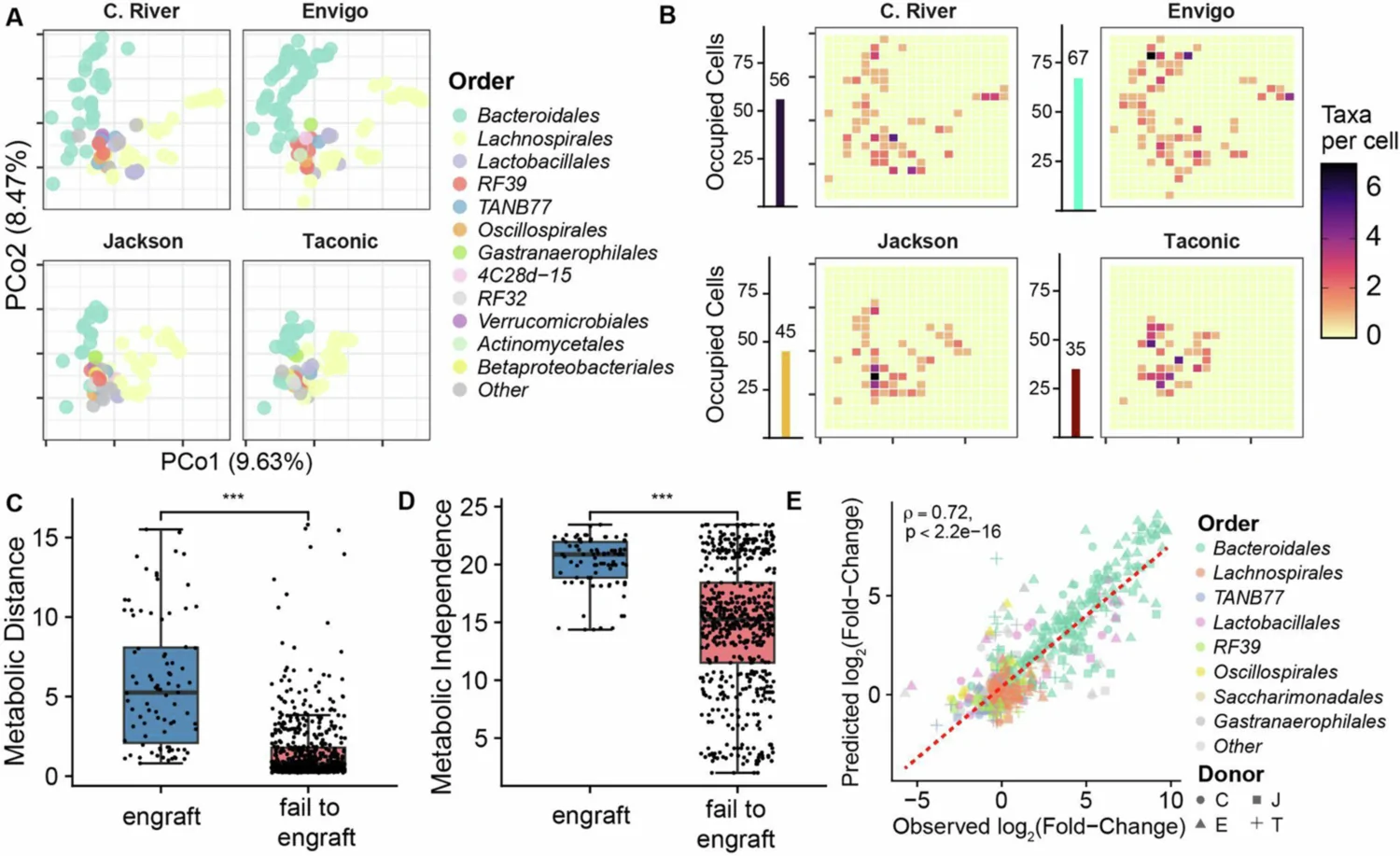
Metabolic distance predicts engraftment outcomes in a murine FMT model. (A) Principal coordinate analysis (PCoA) of MICOM-derived metabolic exchange flux profiles for metagenome-assembled genomes (MAGs) from four vendor-distinct murine microbiomes (Charles River, Envigo, Jackson, and Taconic). Each point represents one MAG, colored by taxonomic order. (B) Grid occupancy of the metabolic niche space. A shared 20 x 20 grid was overlaid on PCoA coordinates across all four communities; tile color indicates the number of taxa per cell. Inset bar plots show the total number of occupied grid cells for each community. (C) Metabolic distance of donor taxa to the recipient community for taxa that were successfully engrafted versus those that failed to engraft (*p* < 2.2 × 10^−16^, two-sided Mann‒Whitney U test). (D) Metabolic independence scores for engrafting versus non-engrafting taxa (*p* < 2.2 × 10^−16^, two-sided Mann‒Whitney U test). (E) Random forest regression predicting post-FMT changes in MAG relative abundance. Each point represents one MAG across all donor‒recipient pairings, colored by taxonomic order and shaped by donor identity. The dashed line indicates the linear fit. The predictions shown are held-out values from 10-fold cross-validation.

We next examined whether the four vendor microbiomes differed in their coverage of the metabolic niche space. We performed PCoA on MICOM-derived exchange flux profiles using cosine distance, which compares the proportional distribution of fluxes across metabolites and is therefore sensitive to differences in metabolic strategy independent of absolute flux magnitude. MAGs clustered largely according to their assigned taxonomic order, yet we also observed substantial variability within each order, indicating that closely related MAGs can possess markedly different predicted metabolic capabilities (Figure 1A).^26^ We then divided the resulting two-dimensional niche space into a 20 × 20 grid to evaluate cell occupancy across microbiomes (see Methods). The Envigo microbiome occupied the greatest number of grid cells (*n* = 67), followed by Charles River (*n* = 56), Jackson Laboratories (*n* = 45), and Taconic (*n* = 35) (Figure 1B). This ranking corresponded to prior observations of engraftment potential in this system, where Envigo was the most successful donor and Taconic the least,^23^ suggesting that broader metabolic niche coverage may confer a colonization advantage.

To formalize this relationship, we developed a quantitative metric of metabolic niche complementarity termed metabolic distance (see Methods). For each donor‒recipient pair, we compared the predicted metabolic flux profiles of the donor and recipient MAGs and summarized these pairwise comparisons into a single, community-level value for each donor MAG, where larger values indicate greater metabolic dissimilarity between a donor MAG and the recipient community. In parallel, we used anvi'o^27^ ^,^ ^28^ to estimate metabolic independence for each MAG, a metric that evaluates the comprehensiveness and completeness of metabolic pathways within genomes, where microbes with more complete pathways are considered more independent. Comparing these values to colonization outcomes, we found that donor MAGs that successfully colonized recipient communities exhibited significantly greater metabolic distance (Figure 1C, *p* < 2.2 × 10^−16^, Mann‒Whitney U test). We evaluated several alternative distance metrics for this comparison, including L1, L2, and cosine distance, all of which separated colonizers from non-colonizers in the same direction but with a smaller effect size than the Mahalanobis distance (Figure S2). Successfully colonizing donor MAGs were also more metabolically independent (Figure 1D, *p* < 2.2 × 10^−16^, Mann‒Whitney U test) than non-engrafting ones.

Given that metabolic distance and metabolic independence were strongly associated with engraftment, we next evaluated whether these features, together with simple community descriptors, could predict colonization outcomes. We trained a random-forest classifier to predict colonization outcomes using metabolic distance, metabolic independence, the pre-FMT abundances of MAGs in donors and recipients, and the ratio of Shannon diversity scores between donors and recipients, which has been previously associated with greater transfer during FMT.^23^ This classifier accurately separated colonizing from non-colonizing taxa across all donor‒recipient pairings (auROC = 0.998; prAUC = 0.795; null prAUC = 0.148, Figure S3A, S3B) and maintained a median auROC of 0.918 even when trained on only 10% of the data (Figure S3C). We used ANOVA to assess the amount of variance explained by each parameter and found that metabolic distance was the strongest predictor of colonization, explaining 41.5% of the variance in colonization outcomes (Figure S3D). Using the same model architecture, we trained a random forest regression model to quantitatively predict changes in MAG relative abundance within FMT recipients. This model predicted post-FMT changes in abundance with an average Spearman rho of 0.718 ± 0.045 accuracy across 10-fold cross-validation (Figure 1E, *p* < 2.2 × 10^−16^). Collectively, these findings show that metabolic distance, derived from community-scale metabolic models, provides a simple yet powerful predictor of which microbes will engraft during FMT and the resulting composition of recipient communities.

### Metagenomic and metabolic signatures of IBS-D/M

Irritable bowel syndrome with diarrhea or mixed stool pattern (IBS-D/M) is a prevalent functional gastrointestinal disorder that imposes substantial costs on patients and health systems.^29^ Multiple studies implicate the gut microbiome in IBS pathophysiology, including alterations in community composition, gas production, and fermentation profiles.^30^ ^,^ ^31^ Microbiome-targeted interventions such as FMT and defined microbial therapeutics have shown promise in subsets of patients, but clinical responses are highly variable, and the microbial or metabolic features that distinguish responders from non-responders remain poorly defined.^32-34^

We reasoned that metabolic modeling could capture key microbial and predicted metabolic signatures associated with IBS pathophysiology and distinguish patients according to their clinical response to FMT, as defined by changes in IBS symptom severity. To test this, we performed a retrospective analysis of a study that assessed the efficacy of FMT in treating IBS-D/M patients.^35^ This study implemented a randomized, double-blind, placebo-controlled, parallel-group, single-center study (*n* = 30) for IBS. The participants met the ROME III criteria for IBS-D or IBS-M, ranged from 18 to 75 years old, and exhibited moderate-to-severe symptoms based on the IBS Severity Scoring System (IBS-SSS ≥ 175). Stool samples were collected for metagenomic sequencing from donors and from participants at baseline (T0), 6-month (T6), and 12-month (T12) follow-ups. At 3 months, patients were reassessed for IBS and deemed “responders” if they achieved a 75-point decrease in IBS-SSS.^36^ Importantly, no antibiotic pretreatment was administered to the study participants prior to FMT. This was intentional, as our framework infers colonization outcomes from donor–recipient metabolic complementarity, which would be obscured by antibiotic depletion of the resident microbiome.

**Figure 2.**
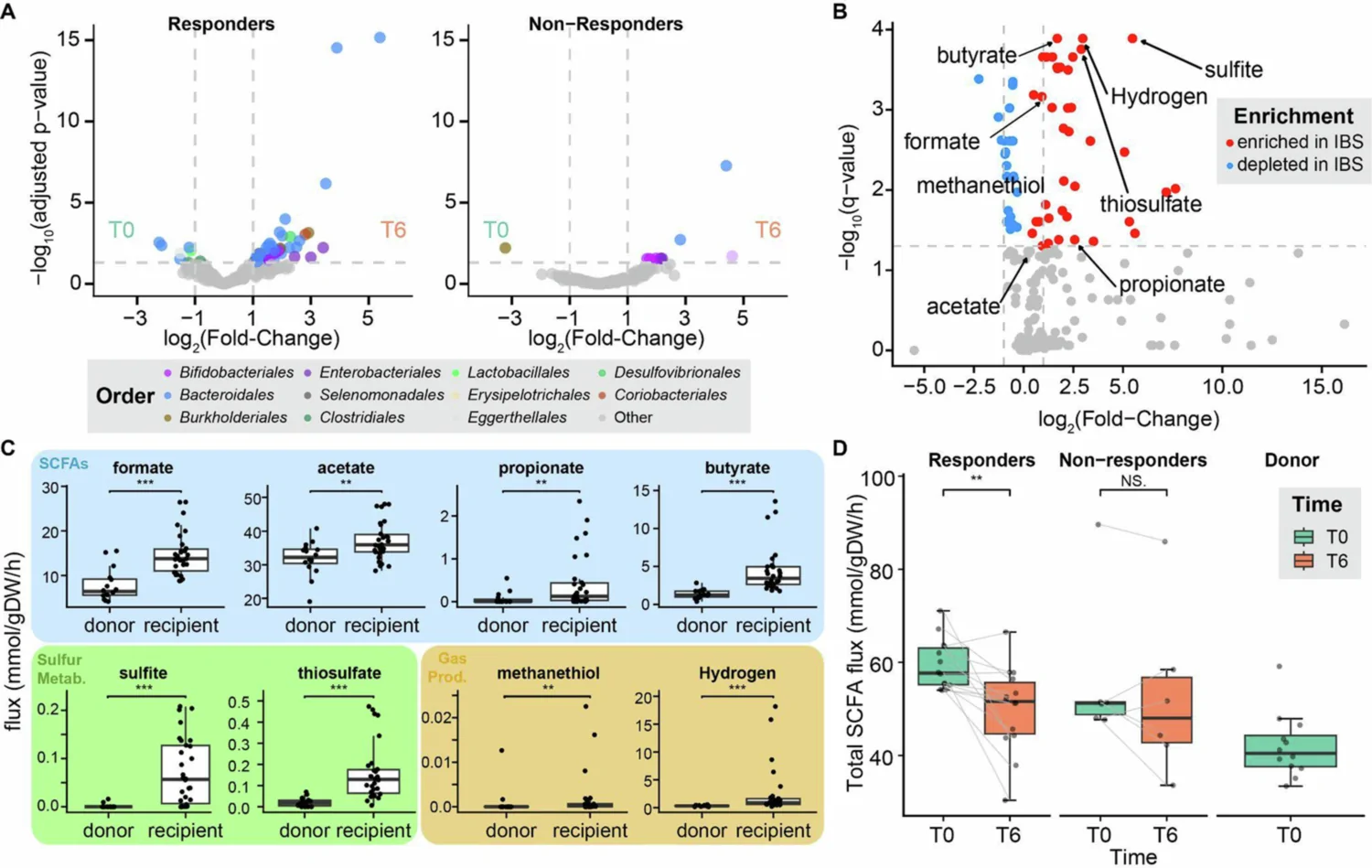
Community-scale metabolic models recapitulate the compositional and metabolic signatures of IBS-D/M. (A) Differential abundance analysis comparing overall recipient microbiome composition between baseline (T0) and 6 months post-FMT (T6) for responders (left) and non-responders (right). Each point represents one taxon, colored by taxonomic order. The x-axis shows the log2-normalized fold change (T6 vs T0), and the y-axis shows -log10 adjusted *p*-value. Responders showed significant enrichment of *Bacteroidales* at T6, while non-responders exhibited minimal compositional change. (B) Differential metabolic flux analysis between IBS patients at baseline and healthy donors, computed from MICOM community-scale metabolic models. Each point represents one secreted metabolite; red indicates metabolites with higher predicted production in IBS patients, and blue indicates metabolites with higher predicted production in donors. Key metabolites are labeled, including SCFAs (butyrate, formate, propionate, acetate), sulfur metabolites (sulfite, thiosulfate), and gas products (hydrogen and methanethiol). The dashed line indicates the significance threshold. (C) Predicted community export fluxes for individual SCFAs (blue, top row), sulfur metabolites (green, bottom left), and gas products (yellow, bottom right) in donors versus IBS recipients at baseline. IBS patients showed significantly elevated production across all metabolite classes. Boxplots show the median and interquartile range with individual data points overlaid; significance was determined by a two-sided Mann‒Whitney U test (**p < 0.01, ***p < 0.001). (D) Total predicted SCFA flux (sum of formate, acetate, propionate, and butyrate) at T0 and T6 for responders, non-responders, and donors. Lines connect paired samples from the same patient. Responders showed a significant decrease in total SCFA production following FMT (*p* < 0.01), whereas the non-responders showed no significant change (NS).

Microbiome analysis revealed significant compositional differences between healthy donors and IBS patients before FMT, including a significant increase in *Bacteroidales* species in donors (Figure S4). To link these patterns to clinical response, patient microbiomes at T6 were compared to their respective T0 baselines, with differences evaluated separately for responders and non-responders while accounting for patient-specific variation across time points. The responders showed strong enrichment of *Bacteroidales* following FMT, whereas non-responders exhibited minimal compositional changes, indicating a positive association between post-FMT *Bacteroidales* enrichment and remission of IBS symptoms (Figure 2A). To assess overall community structure across groups, we performed principal coordinate analysis on Bray‒Curtis dissimilarities of relative abundances for donors, pre-FMT recipients, and post-FMT recipients (Supplementary Figure S5). Donors were separated from recipients along the primary axis both before and after FMT (PERMANOVA, donor vs pre-FMT recipients, R2 = 0.23, *p* < 0.001), indicating that donor and recipient communities occupy distinct regions of composition space.

We then explored whether differences in microbial composition also drove differences in the metabolic activities of the microbiome that could indicate disease. Specifically, we evaluated whether community-level metabolic models could capture these differences and provide insight into the molecular drivers of IBS-D/M development and remission. We constructed community metabolic models by mapping metagenomic reads from the Goll et al. IBS cohort to genomes from the AGORA database,^37^ a set of 818 genomes from human gut microbiomes with curated GEMs. After generating models for all donors and recipients at T0 and T6, for each of the 468 molecular products secreted by each community, we calculated a weighted export flux, which was defined as the sum of the product of the normalized export flux and the relative abundance of each microbe. IBS patient microbiomes showed significantly elevated production of short-chain fatty acids (SCFAs), sulfur metabolites, and gases (e.g., hydrogen and methane) prior to FMT compared to healthy donors (Figure 2B, C). These predicted patterns are broadly consistent with previous clinical and experimental reports of increased SCFA profiles, enhanced sulfur-associated microbial metabolism, and increased hydrogen production in IBS-D patients.^30^ ^,^ ^31^ We also noted substantial heterogeneity across previous reports, with differences in metabolic profiles driven primarily by the IBS subtype.^30^ ^,^ ^31^ Thus, these community-scale models capture metabolic signatures characteristic of IBS-D/M and demonstrated the potential of metabolic modeling to capture subtype-specific metabolic alterations.

Given that our metabolic models recapitulated multiple metabolic alterations previously reported in IBS-D/M patients, we next examined whether predicted SCFA production can serve as a key metabolic readout for remission of IBS following FMT. Using weighted export flux, we defined total SCFA production as the sum of the total flux of propionate, acetate, butyrate, and formate, and compared it between T0 and T6 for this IBS cohort. The responders showed a significant decrease in total SCFA production at T6 relative to their T0 baseline (Mann‒Whitney U test, *p* = 0.0021), reaching levels comparable to those of the donors (Figure 2D). In contrast, non-responders did not exhibit a significant change in predicted total SCFA production following FMT (Mann‒Whitney U test, *p* = 0.7). However, there were no significant overall differences in total SCFA flux between responders and non-responders at T6, suggesting that additional factors, such as non-SCFA metabolites, may drive symptoms in these non-responders to FMT. Together, these results indicate that metabolic modeling captures key compositional and functional features of IBS-D/M and that a successful response to FMT is associated with an increased abundance of *Bacteroidales* and reduced community SCFA production.

### Systematic donor-recipient mapping for predicting post-FMT microbiome composition

Building on our findings that metabolic models predict post-FMT microbiome composition and capture IBS-D/M-associated metabolic signatures, we next asked whether these GEMs could be used prospectively to guide donor selection for FMT. Specifically, we sought to test whether the same modeling framework could be repurposed to identify donor-recipient combinations to maximize post-FMT compositional shifts and metabolic shifts toward a donor-like state.

**Figure 3.**
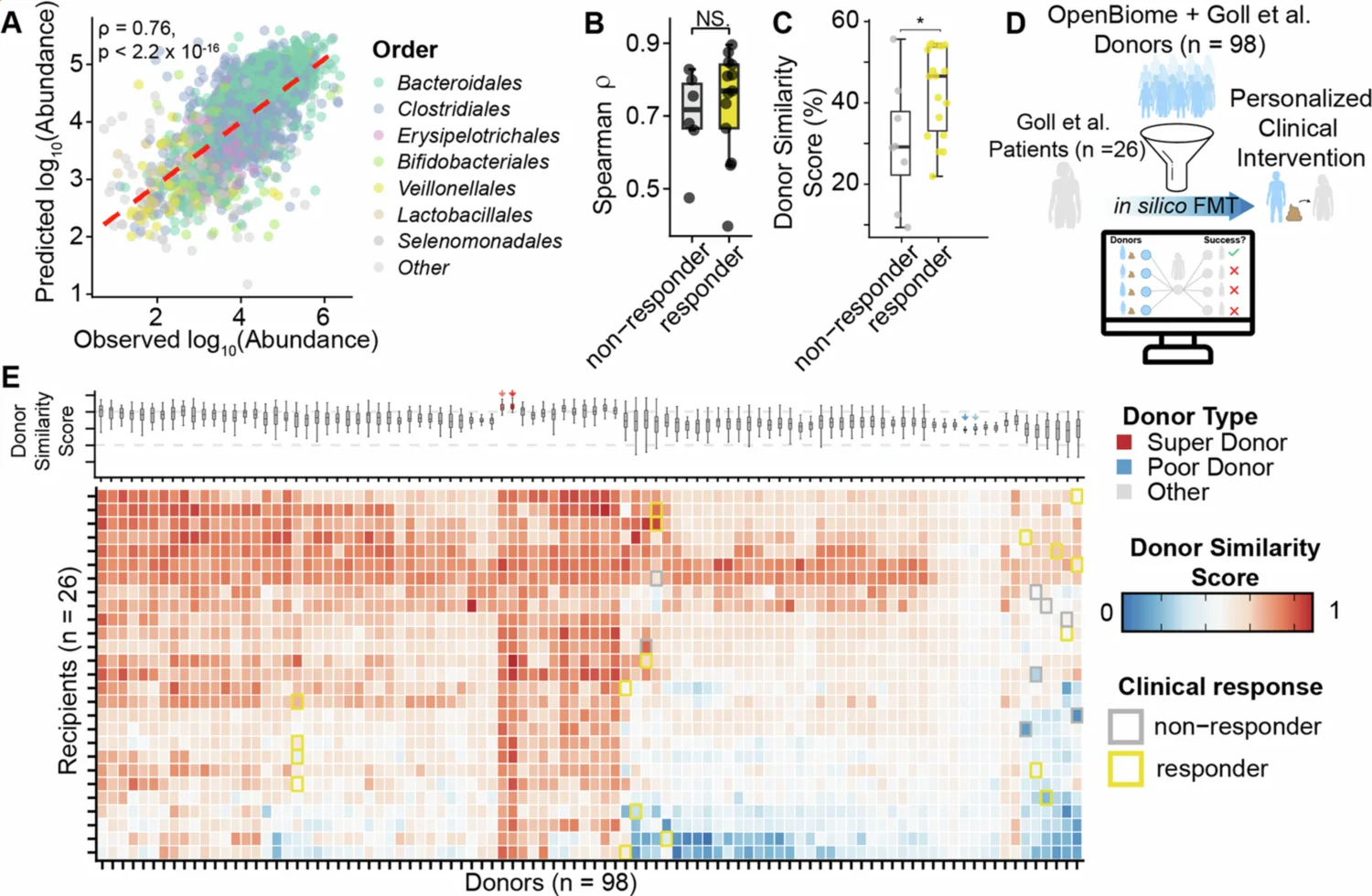
Metabolic models predict post-FMT composition in human IBS and enable *in silico* donor screening. (A) Leave-one-recipient-out cross-validation of a random forest regression model predicting post-FMT taxon abundance from metabolic distance, metabolic independence, pre-FMT abundances, and the donor-to-recipient diversity ratio. Each point represents one taxon for one recipient, colored by taxonomic order. The red dashed line indicates the linear fit; the Spearman rho and *p*-value are annotated. (B) Per-recipient Spearman rho between predicted and observed post-FMT abundance, stratified by clinical response. Each point represents one recipient held out during model training. NS, not significant (Wilcoxon rank-sum test). (C) Donor similarity scores for responders and non-responders, calculated as the relative shift in the Bray‒Curtis distance from the pre-FMT composition toward the donor composition at 6 months post-FMT (*p < 0.05, Wilcoxon rank-sum test). A score of 0% indicates no change in the microbiome, whereas a score of 100% indicates a complete shift to match the composition of the donor. (D) Schematic of the *in silico* FMT screening framework. Metabolic models from 26 Goll et al. IBS patients, 12 Goll et al. donors, and 86 OpenBiome donors were used to simulate 2,548 donor‒recipient pairings and predict post-FMT microbiome composition. (E) Heatmap of predicted donor similarity scores across all donor‒recipient combinations. The rows represent recipients (*n* = 26), and the columns represent donors (*n* = 98), ordered by hierarchical clustering. The color intensity reflects the predicted donor similarity score (red = high, blue = low). Bordered tiles indicate actual donor‒recipient pairings from the Goll et al. trial (gold = responder, gray = non-responder). Boxplots above show the distribution of predicted donor similarity scores across recipients for each donor; arrows indicate super-donors (red) and poor donors (blue).

We first assessed whether our metabolic modeling approach could accurately predict post-FMT microbiome composition in human IBS studies. Using metagenome-scale metabolic models constructed from IBS-D/M patients prior to FMT, we computed the metabolic distance between patients and their actual donors and predicted the composition of patients 6 months after treatment. The predicted and observed post-FMT compositions were strongly concordant across leave-one-recipient-out cross-validation (Spearman rho = 0.76 ± 0.14, *p* < 2.2 × 10^−16^; Figure 3A), and the prediction accuracy was consistent for both responders and non-responders (Figure 3B). To examine whether donor-like compositional changes were associated with clinical remission, we quantified the shift by measuring the reduction in Bray‒Curtis dissimilarity between each recipient and its paired donor following FMT (see Methods). We refer to this metric as the donor similarity score, with higher values indicating greater post-FMT compositional similarity to the paired donor. By this metric, clinical responders shifted significantly closer to their donors than non-responders (*p* < 0.05, Wilcoxon rank-sum test; Figure 3C), indicating that responder microbiomes became more compositionally similar to their paired donors following treatment than non-responder microbiomes. Together, these results demonstrate that metabolic distance-based models capture microbiome features that predict post-FMT community composition in human cohorts and that donor-like compositional shifts are associated with improved clinical outcomes.

We then asked whether a model trained to predict post-FMT microbiome composition could serve as a practical platform to screen and select donors for personalized FMT. We used the same modeling framework to perform *in silico* FMT simulations for 2,548 FMTs, representing all pairwise combinations of 26 IBS-D/M patients and 98 candidate donors, including 12 from the original Goll et al. cohort and 86 individuals characterized by the OpenBiome project (Figure 3D). This analysis revealed substantial heterogeneity in the predicted post-FMT microbiome composition across donor‒recipient pairs, driven by both donor and recipient identities (Figure 3E). To determine whether donor or recipient identity contributed more strongly to post-FMT microbiome composition, we subsampled 50% of all simulated donor‒recipient combinations across 100 iterations and calculated the proportion of total variance in the donor similarity score explained by each factor. Recipient identity explained a median of 41.7% of the variance compared with 34.9% for donor identity, indicating that recipient-specific features contributed modestly more in determining the donor-like compositional shift (Wilcoxon rank sum test, *p* < 2.2 × 10^−16^). Current approaches to FMT donor selection focus on identifying universal “super-donors” while remaining largely agnostic to recipient characteristics, yet these results suggest that matching donors to recipients based on recipient-specific features may be more effective than optimizing donor selection alone.

### Community characteristics of “super-donor” microbiota

**Figure 4.**
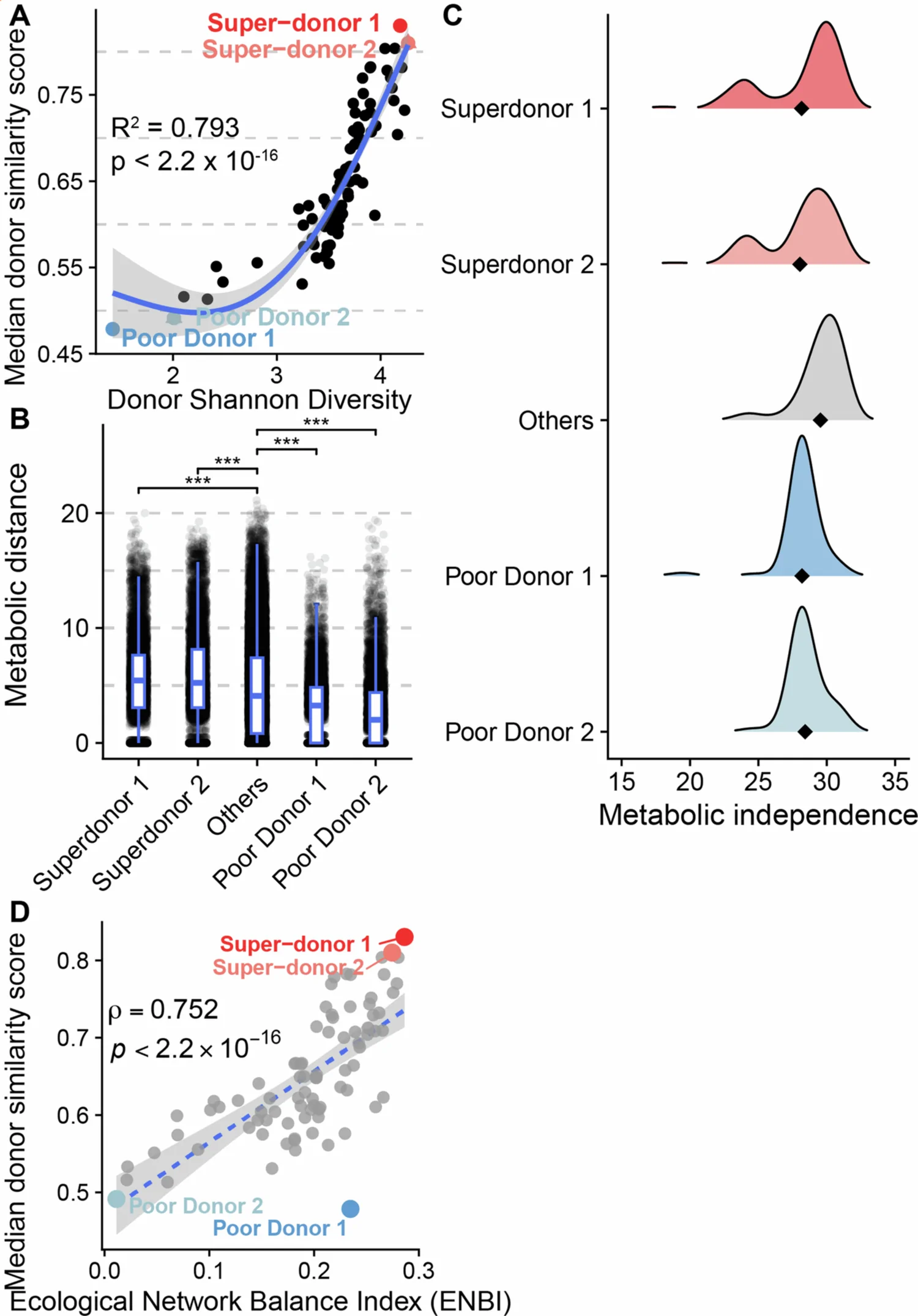
Community ecological characteristics of super-donors. (A) Relationship between donor Shannon diversity and the median predicted donor similarity score across all recipients. Each point represents one donor; the blue line shows a generalized additive model (GAM) fit with a 95% confidence interval (R^2^ = 0.793, *p* < 2.2 × 10^−16^). Super-donors 1 and 2 (red) occupy the high-diversity, high-similarity extreme; poor donors 1 and 2 (blue) cluster at the low end. (B) Distribution of metabolic distance to recipients for taxa from super-donors, other donors, and poor donors. Super-donor taxa exhibited significantly greater metabolic distance from recipient communities than taxa from other donors (***p < 0.001, Mann‒Whitney U test). Boxplots show the median and interquartile range with individual data points overlaid. (C) Density distributions of metabolic independence scores for taxa within each donor category. The diamonds indicate the median. The super-donor communities displayed a bimodal distribution of metabolic independence, with a subpopulation of low-independence taxa absent in poor donors. (D) Relationship between the ecological network balance index (ENBI), computed from MICOM-derived pairwise metabolic fluxes, and the median donor similarity score across all donors. Each point represents one donor, with super-donors (red) and poor donors (blue) labeled. The ENBI quantifies the relative dominance of positive (cross-feeding) over negative (competitive) interactions within each donor community (Spearman rho = 0.752, *p* < 2.2 × 10^−16^). The dashed line shows the linear fit with a 95% confidence interval.

While the existence of “super-donors” in FMT has been suggested,^11^ ^,^ ^38^ the community ecological characteristics have not been deeply investigated. Although recipient identity generally exerted a greater influence on predicted post-FMT compositions than donor identity, two donors appeared to be notable exceptions, exhibiting consistently high predicted donor compositional convergence across nearly all simulated recipients. These donors, henceforth referred to as “super-donors” 1 and 2, consistently achieved the highest donor similarity scores, with median values of 0.83 ± 0.064 and 0.81 ± 0.059, respectively. To shed more light on the community ecological characteristics of super-donor microbial communities, we performed a series of community analyses, contrasting with two “poor donors”, which exhibited the lowest average donor similarity scores across the simulated transplants. The super-donors harbored some of the highest Shannon diversity values among all the donors, which strongly correlated with median donor-similarity scores across the cohort (Figure 4A, R^2^ = 0.793, *p* < 2.2 × 10^−16^, GAM). Donor *α*-diversity has previously been reported to be positively associated with donor strain engraftment across 14 FMT trials.^11^ Moreover, taxa from super-donors were, on average, more metabolically distant from recipients than taxa from other donors (Figure 4B, *p* < 2.2 × 10^−16^, Mann‒Whitney U test), consistent with our model that complementary, orthogonal metabolisms promote compositional convergence. Analyzing the distribution of metabolic independence scores within these communities, the super-donors were less metabolically independent on average (*p* < 2.2 × 10^−16^, Mann‒Whitney U test; Figure 4C), with differences largely attributable to a bimodal distribution in metabolic independence scores that was absent in poorly performing donors. This bimodal distribution suggested a community composed of a mixture of metabolically self-sufficient and cross-feeding-dependent taxa, implying extensive within-community metabolic cooperation.

The reduced average metabolic independence of super-donor communities implies that their taxa are substantially reliant on cross-feeding to meet their metabolic demands. Corral Lopez et al.^39^ showed that tightly cross-feeding consortia form compact metabolic loops that efficiently exploit available resources, allowing them to outcompete and displace resident taxa. We reasoned that this dynamic may similarly operate in the FMT context, where super-donor consortia with analogous cross-feeding architectures could more effectively overcome colonization resistance in the recipient gut. Inspired by the ecological network balance index (ENBI) by Corral Lopez et al.^39^, which quantifies the relative balance of positive and negative interactions within a community, we computed an analogous metric using MICOM-derived metabolic fluxes to evaluate pairwise competitive and cross-feeding interactions among all donor community members (see Methods). This analysis showed that flux-derived interaction balance correlated strongly with donor similarity score (Figure 4D, *ρ* = 0.752, *p* < 2.2 × 10^−16^), suggesting that the cross-feeding network architecture characteristic of super-donor communities may provide a mechanistic basis for the super-donor phenomenon by facilitating greater compositional convergence associated with clinical remission following FMT.

### Generalization of metabolic predictors across FMT cohorts

To assess whether this framework generalizes beyond the cohort in which it was trained, we applied it to an independent FMT trial in which recipients received either allogeneic or autologous transplants, without antibiotic treatment prior to FMT, and were sampled at four time points through 84 d (*n* = 10 recipients, 5 allogeneic and 5 autologous).^40^ Trained and evaluated entirely within this cohort by leave-one-recipient-out cross-validation, the model predicted post-FMT composition with accuracy comparable to the IBS cohort (*ρ* = 0.78 ± 0.08, *p* < 2.2 × 10^−16^; Figure S6A). Pre-FMT abundance carried the greatest feature weight in this cohort, consistent with a trial design in which half of the recipients received their own microbiota (Figure S6B). When the model trained on the IBS cohort was instead applied to these patients without retraining, the predicted fold-changes correlated only weakly with the observed changes at every timepoint (median *ρ* = 0.20, 0.10, 0.04, and 0.08 at days 2, 14, 42, and 84, respectively), which was well below the fold-change accuracy that the same model achieved within the IBS cohort itself (Figure S6C). These results indicate that metabolic features remain informative in an independent cohort, but that a predictor trained on a single trial does not transfer directly to a cohort differing in indication, transplant route, or sampling interval, and that broader multi-cohort training sets will be needed to build generalized predictors of FMT outcome.

## Discussion

In this study, we used metagenome-scale metabolic modeling to show that the ecological theory of niche complementarity shapes donor microbial engraftment and informs more rational donor selection strategies in FMT. By leveraging MICOM-based community models and a new metric of metabolic distance, we showed that donor taxa that are more metabolically dissimilar from the recipient microbiome are more likely to engraft in a murine FMT model. Metabolic distance and metabolic independence together explained a substantial fraction of engraftment variance and enabled accurate prediction of colonization outcomes using simple machine learning models. We then extended this framework to a human IBS-D/M FMT trial, where community-scale models recapitulated disease-associated metabolic alterations and identified distinct metabolic features associated with the clinical response to FMT. Finally, we demonstrated how this framework can be used to systematically evaluate donor‒recipient compatibility through large-scale *in silico* FMT simulations by leveraging model-derived features to predict post-FMT community compositional shifts. Across all donors, we identified two super-donors whose taxa occupied a broader region of metabolic niche space, achieved consistently high predicted donor similarity scores across diverse recipients, and provided a functional explanation for the super-donor phenomenon. Together, these results support metabolic niche complementarity as a determinant of donor microbial colonization and of predicted shifts in microbiome metabolic output.

Our findings build upon existing models for predicting FMT success that have focused primarily on taxonomic or pathway composition, phylogenetic relatedness, or simple diversity metrics.^7^ ^,^ ^8^ ^,^ ^10^ Most comprehensively, Podlesny et al. applied strain-resolved metagenomics across 14 FMT trials to identify clinical and ecological determinants of engraftment, demonstrating that donor strain engraftment depends on pre-FMT recipient dysbiosis, donor and recipient *α*-diversity, and species-level traits, including relative abundance and oxygen tolerance, and that models built over simulated donor‒recipient pairings could, in principle, guide personalized donor selection.^11^ Additional prior work has shown that donor‒recipient similarity in community structure or the presence of particular taxa can correlate with FMT outcomes, yet these features are defined at the level of taxonomy and community structure and do not directly capture how communities use resources or partition metabolic functions.^10^ In contrast, community metabolic modeling can explicitly represent resource competition and cross-feeding, enabling ecological interpretations between donor and recipient communities to be quantified rather than inferred from simple metrics. Here, we show that a single model-derived feature, metabolic distance, is sufficient to accurately predict which taxa will colonize and their relative abundances after transplantation. This is consistent with ecological theory in which successful invaders occupy underutilized niches and face less direct competition from resident species.^15^ ^,^ ^16^ By embedding high-dimensional flux profiles into an interpretable niche space, our framework moves beyond black-box correlational models and provides a mechanistic link between metabolic complementarity and colonization resistance.

These insights could form the basis for FMT donor selection for IBS if proven on independent, larger cohorts, with clinical response data available. The two super-donors identified in our i*n silico* screen were characterized by high taxonomic diversity, broad metabolic niche coverage, and a bimodal distribution of metabolic independence scores, reflecting metabolically diverse communities composed of both self-sufficient taxa and cross-feeding-dependent specialists. Consistently, a flux-derived analog of ENBI^39^ scored highest in super-donors, suggesting that cross-feeding-dominated interaction networks confer a colonization advantage by enabling tightly cooperative consortia to exploit underutilized metabolic niches in the recipient gut. Notably, Corral López et al. found that elevated ENBI values characterize dysbiotic rather than healthy communities, where tightly cross-feeding consortia outcompete resident taxa and dominate the community. Our results suggest that this same competitive mechanism may operate during FMT, in which incoming donor consortia with strong internal cross-feeding networks can similarly overcome colonization resistance in the recipient. These findings suggest that donor screening should move beyond diversity and taxonomic criteria to explicitly quantify metabolic niche coverage and interaction networks, metrics that can be computed prospectively from metagenomic data. Notably, because these metrics are derived from the intact resident community, donor matching can be performed without antibiotic conditioning of the recipient, which has been shown to increase gut oxygen levels and may inhibit transplantation of strict anaerobes.^41^ ^,^ ^42^ Importantly, because recipient identity explained modestly more variance in FMT compositional outcomes than donor identity, effective donor matching will require joint consideration of the donor's niche profile and the recipient's existing metabolic landscape.

Our retrospective analysis of an IBS-D/M FMT trial captured known features of IBS, including altered SCFA, sulfur, and gas metabolism, and showed that responders to FMT shifted toward donor-like metabolic states. Although greater donor-like compositional shifts trended to be associated with a successful response to FMT, several patients with high donor similarity scores did not experience remission of IBS symptoms.^12^ This incomplete link between engraftment and clinical response has been previously observed,^8^ ^,^ ^43^ and suggests that donor similarity alone is not sufficient and that donor selection should prioritize microbiomes predicted to actively restore disease-associated metabolic functions. With further refinement of environmental priors, metagenome-scale models may be able to predict these effects, enabling FMT strategies that are guided not only by predicted donor–recipient compatibility but also by anticipated changes in microbiome metabolic phenotypes. Such an approach could support targeted and personalized care across diverse microbiome-associated diseases.

Despite these findings, several limitations provide opportunities to further develop and validate this framework both computationally and experimentally. While our models use curated GEMs and metagenomic data, they still rely on steady-state assumptions, standardized environmental conditions, and incomplete knowledge of species-level variations in metabolism, which could potentially influence metabolic distance calculations relative to recipient microbiota. Integrating time-resolved data, diet records, and host-specific constraints could improve the prediction of post-FMT compositional shifts while enabling more accurate estimation of downstream metabolic and clinical responses. Importantly, in cases where microbes shared by the donor and recipient increase following FMT, these increases cannot always be attributed to donor engraftment, but may rather indicate an expansion of native communities. Future FMT studies combining dense longitudinal sampling with deep, long-read sequencing and strain-resolved metagenomics will be essential to definitively quantify strain engraftment. Likewise, integrating metabolomic profiling with community metabolic modeling will allow for models that capture the unique biochemical properties of individuals’ gut environments for incorporating into model priors. Applying this framework to rationally designed consortia^20^ would allow direct experimental evaluation of whether engineered niche complementarity can match or surpass the performance of natural donors. Finally, extending these ideas to additional diseases and patient populations beyond IBS-D/M will be essential for establishing metabolic niche complementarity as a general principle for effective microbiome-based treatments.

## Methods

### Study design and cohorts

Three shotgun metagenomic datasets were analyzed in this study. The first comprised murine fecal samples from C57BL/6 mice sourced from four vendors: Taconic Biosciences, Envigo, Jackson Laboratories, and Charles River, that were characterized in a previous study.^23^ Eight mice per vendor were used, and cohousing was performed in pairs, with each mouse participating in two pairwise cohousings. Samples collected before and after all pairwise cohousings were included, enabling analysis of both vendor-associated microbiome differences and inter-vendor microbiome transfer dynamics.

The second dataset was derived from a human clinical trial (Goll et al. 2020), in which participants with irritable bowel syndrome (IBS) underwent fecal microbiota transplantation (FMT). Among the 83 participants enrolled in the REFIT study, only those in the active treatment arm with complete sample sets were included, yielding 22 participants whose stool was collected at three time points. The participants were stratified by treatment outcome into responders (*n* = 14) and non-responders (*n* = 8). For the *in silico* FMT simulations, an additional four patients were included, for a total of 26, although these individuals did not return for 6-month and 12-month follow-ups, so they were excluded from previous analyses.

A third dataset used in this work was from the Openbiome dataset,^44^ comprising stool samples from healthy donors screened by OpenBiome, a non-profit stool bank. From the full cohort of 90 subjects sampled, the subset of 86 donors for whom shotgun metagenomic sequencing data were available was used in the present analysis. The donors were healthy adults aged 19–45 y, free of gastrointestinal, autoimmune, cardiovascular, neurological, and metabolic conditions at recruitment, screened for pathogens, and fully deidentified prior to sample receipt.

### Metagenomic sequencing, processing, and abundance profiling

Read trimming, adapter removal, and quality filtering were performed using fastp^45^ (v1.0.1) with polyG and polyX tail trimming enabled (-g -x) and a custom adapter FASTA supplied via --adapter_fasta. Taxonomic classification of the cleaned reads was performed using Kraken2^46^ (v2.17.1) against the standard 8 GB database, followed by abundance re-estimation at the species level using Bracken; per-sample Bracken reports were subsequently merged into a single species-level count table. Read identifiers classified as belonging to AGORA-relevant taxa were extracted by cross-referencing Kraken2 output against a predefined ancestral taxa lineage list using csvgrep and csvcut, and matching reads were isolated from the cleaned FASTQ files using a custom filtering script. Filtered reads were then aligned to an indexed AGORA genome database using minimap2^47^ in short-read mode (-x sr) with secondary alignments suppressed (--secondary no). The resulting alignments were filtered using samtools (v1.21) to retain only properly paired, first-in-pair reads mapped in the correct orientation (flags -f 0 × 42 -F 0xF0C), and per-taxon read counts were extracted from the alignment via an inline Perl script parsing the taxon ID from the reference sequence name. The final per-sample count files were aggregated into a single count matrix using a custom Python script. All parallelizable steps were distributed across samples using GNU Parallel (v20250422).

### Genome-scale metabolic model reconstruction and community modeling (CarveMe, MICOM)

The mouse data described in this study were acquired from a publicly available dataset, in which the raw metagenomic sequencing data were processed under a standardized pipeline to generate individual contig assemblies, abundance profiles, and taxonomic annotations.^23^ Unlike human gut microbiomes, for which AGORA provides a standardized collection of pre-built genome-scale metabolic models, no equivalent reference database currently exists for murine microbiomes. Therefore, the assemblies from this study were further processed by CarveMe v1.6.0^24^ with gapfilling to generate the reference model database GEMs before being incorporated into MICOM for community metabolic modeling. For each mouse cohort, namely, Jackson, Envigo, Taconic, and Charles River, a pre-FMT and a post-FMT model were constructed using MICOM v0.37.0 with “cutoff = 0”.^19^ Read counts were averaged within each cohort, and taxonomic information was summarized to the genus level to match the reference database prior to model construction. Raw metagenomic read counts were normalized by genome length to account for read counts for a given taxon being proportional to both its cellular abundance and its genome length. Community metabolic models were constructed from these genus-level abundances. Growth was simulated using the AGORA high-fiber diet ( https://github.com/micom-dev/media ) with “tradeoff = 0.7”.

Abundance and taxonomy data derived from the human clinical IBS FMT treatment data and healthy Openbiome donor data were summarized to the species level. Prior to model construction, for each sample, read counts from each species were further normalized by their respective genome lengths to control for bias caused by uneven sequencing coverage. Model construction was performed at the species level with “cutoff = 0.001”. The predicted metabolic fluxes and growth rates were generated using MICOM’s *grow* function with “tradeoff = 0.7” and a standard AGORA western diet ( https://github.com/micom-dev/media ).

Pairwise metabolic interaction profiles were derived from the resulting community models using MICOM’s interactions function and were used for downstream metabolic distance calculations.

### Metabolic niche space and grid occupancy

MICOM exchange fluxes (day 0, high-fiber diet) were used to place every taxon from every community into a single shared metabolic niche space. Fluxes were rescaled by taxon relative abundance and multiplied by the median abundance across the dataset, yielding per-biomass metabolic profiles that are independent of a taxon's abundance in its community. The absolute flux values were arranged into a matrix in which each row is one taxon in one sample, and each column is one metabolite by direction pair (import or export). Features exchanged by fewer than 1% of taxon by sample rows were removed, zeros were assigned a pseudocount equal to 1% of the smallest observed non-zero flux, and the matrix was log transformed. Pairwise cosine distances (1 minus cosine similarity) were computed across all rows in this matrix, which compares the proportional distribution of flux across metabolites and is therefore sensitive to metabolic strategy rather than total flux magnitude. Principal coordinate analysis with Cailliez correction was applied to this global distance matrix, so that all the taxa from all the communities were embedded in one common set of axes. To quantify niche coverage, the PCo1 by PCo2 plane was divided into a 20 × 20 grid whose bounds were defined by the global range of all taxa across all communities, padded by 1%. For each community, we recorded occupancy, the number of grid cells containing at least one taxon, and density, the number of taxa per occupied cell. Because the ordination and the grid are shared, occupancy and density are directly comparable across communities. Per-cell densities were compared between communities by the Wilcoxon rank-sum test over cells occupying at least one community.

### Metabolic feature engineering (metabolic distance, metabolic independence, and diversity)

The same metabolic framework was applied to both murine and human datasets. The primary difference was the taxonomic resolution, which was summarized at the genus or species level for the murine and the human datasets, respectively. In addition, interaction profiles from the murine dataset were aggregated across biological replicates within each cohort, whereas human samples were analyzed independently without replicate aggregation. These interaction profiles were summarized by metabolite and interacting microbial groups to generate a metabolite-level representation for each microbial genus (mouse analysis) or species (human analysis). Profiles were then reshaped into taxon by metabolite matrices for each donor or recipient community. The pairwise distance was computed between every donor and recipient genus (mouse analysis) or species (human analysis) using the “pairwise_distance” function from scikit-learn. In the primary analysis, the Mahalanobis distance was used because it accounts for the covariance structure of the metabolite space, given the highly correlated nature of exchange fluxes across metabolic pathways, thus preventing correlated fluxes from disproportionately inflating distance estimates. The covariance matrix was estimated using a Ledoit‒Wolf shrinkage estimator to ensure numerical stability given the high dimensionality of the flux data relative to the number of taxa. The resulting donor taxon by recipient taxon matrix captures the metabolic dissimilarity of every donor–recipient pair such that each donor taxon is described by a distribution of distances across the entire recipient community. To summarize this distribution as a single value per donor taxon, we computed the mean distance to its 10 nearest recipient taxa, which represent the organisms occupying the most similar metabolic niches and therefore their most likely niche competitors. Using a fixed neighborhood size avoided dependence on the total number of taxa present within each recipient community while capturing the local metabolic niche surrounding each donor organism. Metabolic independence was computed for each AGORA genome using the anvi-script-estimate-metabolic-independence function in anvi'o v8.0,^28^ and the genome score was extracted.

### Engraftment and outcome definitions (colonization, composition shift, and clinical response)

Colonization was determined using differential abundance analysis. In the murine FMT dataset, individual recipient mice (*n* = 4 per cohort) were treated as biological replicates, and their relative abundance profiles were analyzed using DESeq2 by comparing their microbiomes before and after FMT. Donor-associated genus-level MAGs were subsequently identified as colonizing or non-colonizing based on the following criteria: (i) they were present in the recipient cohort with a mean relative abundance below 0.1%; (ii) they were present in the donor cohort with a mean relative abundance exceeding 0.1%; (iii) they exhibited a significant increase in abundance following FMT (DESeq2 adjusted *p* < 0.05 and log2-fold change > 0); and (iv) they reached a mean relative abundance greater than 0.5% in recipient mice following FMT.

In the human dataset, raw abundance counts were first normalized by taxon-specific genome length and then converted to relative abundance within each sample. Only taxa present in at least 5% of the samples with relative abundance exceeding 0.01% were retained. The raw count matrix was then filtered to retain only this subset of taxa for differential abundance analysis, which was performed to assess T0-to-T6 compositional shifts separately in responders and non-responders using DESeq2 with the formula “~subject_id + time” to control for patient and time effects.^48^

We quantitatively defined “engraftment” as a “donor similarity score”. Bray‒Curtis dissimilarity was calculated between (i) the recipient microbiome at baseline (T0) and its paired donor; (ii) between the recipient microbiome post-FMT (T6) and the same paired donor. The donor-like compositional shift was then defined as the fractional reduction in dissimilarity:

$$\frac{\mathrm{BC}\left(T0,\;D\right)-\mathrm{BC}\;(T6,D)}{\mathrm{BC}\;(T0,\;D)}$$

where BC(T0,D) and BC(T6,D) denote the Bray‒Curtis dissimilarity between the recipient and its paired donor before and after FMT, respectively. For this analysis, the abundance profile was normalized by genome length and converted to relative abundance, but was not subjected to the filtering criterion described above. Normalization by genome length is necessary, as raw metagenomic read counts for a given taxon are proportional to both its cellular abundance and its genome length. Metabolic differential enrichment analysis was performed by first converting MICOM’s flux dataframe into sample-level production rates using MICOM’s production_rates function, followed by MICOM’s compare_group function.

### Predictive modeling and model evaluation (classification, regression, CV, ANOVA/importance)

To predict the colonization outcome of individual donor taxa following FMT, a random forest classifier was trained using the ranger package (R) with five metabolic features calculated for each genome: metabolic distance, metabolic independence, donor taxon relative abundance at baseline (log₁₀-transformed), recipient genome abundance at day 0, and the donor-to-recipient Shannon diversity ratio for the whole microbiome. The classifier was trained with 50 trees and mtry = 2, and the colonization status of each genome was predicted as a binary outcome. Model performance was evaluated using 10-fold cross-validated AUROC computed via the pROC package, and robustness was further assessed through Monte Carlo cross-validation across 19 iterations at training set proportions ranging from 5% to 90%. Precision-recall performance was evaluated over 20 iterations at a 30/70 train/test split using the PRROC package, with a null model generated by label permutation as a baseline comparator. Feature importance was assessed using the Gini impurity criterion. A complementary random forest regression model was additionally trained to predict the magnitude of colonization as log-transformed fold change, using 150 trees with permutation-based importance, and was evaluated using 10-fold cross-validated R^2^, Pearson correlation, and Spearman rank correlation between observed and cross-validated predicted values.

### *In silico* donor-recipient screening and super-donor analyses

OpenBiome donor samples (BioProject: PRJNA544527, subset of whole-genome shotgun samples) were downloaded and processed as described above for metagenomic sequencing processing and mapping to AGORA reference with paired GEMs. Community-scale metabolic models were reconstructed using MICOM for each OpenBiome donor, and community metabolic flux profiles were generated as previously described. For each donor taxon, metabolic independence scores were computed using anvi'o v8.0, metabolic distance was calculated for each donor taxon relative to each Goll et al. recipient community at baseline, and the donor and recipient taxon abundances at T0 were extracted from their respective AGORA-mapped count tables. Shannon diversity scores were computed for all OpenBiome donor microbiomes and all Goll et al. recipient microbiomes at T0, and donor-to-recipient diversity ratios were derived as described above. The random forest classifier trained on the Goll et al. human FMT dataset was then applied to all OpenBiome donor–recipient pairs to predict post-FMT colonization outcomes, enabling *in silico* screening of donor‒recipient compatibility across the full OpenBiome cohort.

### Evaluation of metabolic competition and cross-feeding

Species-level pairwise interaction estimates were obtained using MICOM's summarize_interactions function, which classifies each metabolic exchange as co-consumed (competition), provided, or received (cross-feeding). For each sample, we summed the total flux across all co-consumed interactions (competition) and across all provided and received interactions (cross-feeding). We then calculated the net interaction balance (rho) as the normalized difference between total cross-feeding flux and total competition flux: rho = (total cross-feeding flux – total competition flux)/(total cross-feeding flux + total competition flux). This metric ranges from −1 (entirely competition-dominated) to +1 (entirely cross-feeding-dominated) and follows the formulation of Corral Lopez et al.^39^

## Supplementary Material

SupplementaryFigures.docx

## Data Availability

The datasets generated and/or analyzed during the current study are available at https://github.com/urtecholabucsd/FMT. Raw data for OpenBiome samples were downloaded from BioProject: PRJNA544527. The raw data for the Goll et al. 2020 FMT trial were downloaded from the European Nucleotide Archive (ENA) under the study accession number PRJEB36140. The raw data used for murine FMT analysis are available through NIH Bioproject: PRJNA1028308. Raw data for Li et al. 2016 samples were downloaded from BioProject: PRJEB12357.

## Statistical analysis, software, and reproducibility

Data handling was performed with Python (3.9.0) using pandas (v2.3.3) and numpy to generate CSV files for downstream statistical analyses and visualizations in R (v4.5.1) using ggplot2 (v4.0.2) and geom_signif extension. Beta diversity analysis was performed with skbio (v0.7.0) in Python. The Mahalanobis distance was calculated using scikit-learn (v1.5.1). Significance in metabolic production rates between donors and recipients at T0, compositional shifts across all donor‒recipient combinations, and changes in total SCFA production rates between responders and non-responders relative to their T0 baseline values were determined by two-sided Mann‒Whitney U tests. For multi-group comparisons, *p*-values were adjusted using the false discovery rate method. Reproducible workflows were implemented through Jupyter Notebooks and R scripts and are available at https://github.com/urtecholabucsd/FMT.

## References

[1] Duvallet C , Gibbons SM , Gurry T , Irizarry RA , Alm EJ . Meta-analysis of gut microbiome studies identifies disease-specific and shared responses. Nat Commun. 2017;8:1784. doi: 10.1038/s41467-017-01973-8. 29209090 PMC5716994

[2] Feuerstadt P , Louie TJ , Lashner B , Wang EE , Diao L , Bryant JA , Sims M , Kraft CS , Cohen SH , Berenson CS , et al. SER-109, an oral microbiome therapy for recurrent infection. NEJM. 2022;386:220–229. doi: 10.1056/NEJMoa2106516. 35045228

[3] Vrieze A , Van Nood E , Holleman F , Salojärvi J , Kootte RS , Bartelsman JF , Dallinga–Thie GM , Ackermans MT , Serlie MJ , Oozeer R , et al. Transfer of intestinal microbiota from lean donors increases insulin sensitivity in individuals with metabolic syndrome. Gastroenterology. 2012;143:913–6.e7. doi: 10.1053/j.gastro.2012.06.031. 22728514

[4] Proença IM , Allegretti JR , Bernardo WM , de Moura DT , Ponte Neto AM , Matsubayashi CO , Flor MM , Kotinda AP . Fecal microbiota transplantation improves metabolic syndrome parameters: systematic review with meta-analysis based on randomized clinical trials. Nutr Res. 2020;83:1–14. doi: 10.1016/j.nutres.2020.06.018. 32987284

[5] Porcari S , Benech N , Valles-Colomer M , Segata N , Gasbarrini A , Cammarota G , Sokol H , Ianiro G . Key determinants of success in fecal microbiota transplantation: from microbiome to clinic. Cell Host Microbe. 2023;31:712–733. doi: 10.1016/j.chom.2023.03.020. 37167953

[6] Deng L , Wang S . Colonization resistance: the role of gut microbiota in preventing invasion and infection. Gut Microbes. 2024;16:2424914. doi: 10.1080/19490976.2024.2424914. 39514544 PMC11552263

[7] Smillie CS , Sauk J , Gevers D , Friedman J , Sung J , Youngster I , Hohmann EL , Staley C , Khoruts A , Sadowsky MJ , et al. Strain tracking reveals the determinants of bacterial engraftment in the human gut following fecal microbiota transplantation. Cell Host Microbe. 2018;23:229–240.e5. doi: 10.1016/j.chom.2018.01.003. 29447696 PMC8318347

[8] Ianiro G , Punčochář M , Karcher N , Porcari S , Armanini F , Asnicar F , Beghini F , Blanco-Míguez A , Cumbo F , Manghi P , et al. Variability of strain engraftment and predictability of microbiome composition after fecal microbiota transplantation across different diseases. Nat Med. 2022;28:1913–1923. doi: 10.1038/s41591-022-01964-3. 36109637 PMC9499858

[9] Shtossel O , Turjeman S , Riumin A , Goldberg MR , Elizur A , Bekor Y , Mor H , Koren O , Louzoun Y . Recipient-independent, high-accuracy FMT-response prediction and optimization in mice and humans. Microbiome. 2023;11:181. doi: 10.1186/s40168-023-01623-w. 37580821 PMC10424414

[10] Schmidt TSB , Li SS , Maistrenko OM , Akanni W , Coelho LP , Dolai S , Fullam A , Glazek AM , Hercog R , Herrema H , et al. Drivers and determinants of strain dynamics following fecal microbiota transplantation. Nat Med. 2022;28:1902–1912. doi: 10.1038/s41591-022-01913-0. 36109636 PMC9499871

[11] Podlesny D , Durdevic M , Paramsothy S , Kaakoush NO , Högenauer C , Gorkiewicz G , Walter J , Fricke WF . Identification of clinical and ecological determinants of strain engraftment after fecal microbiota transplantation using metagenomics. Cell Rep Med. 2022;3:100711. doi: 10.1016/j.xcrm.2022.100711. 35931074 PMC9418803

[12] Behling AH , Portlock T , Ho D , Wilson BC , Paramsothy S , Kamm MA , Cutfield WS , Kaakoush NO , O’Sullivan JM , Vatanen T . Cohort-specific determinants of donor strain engraftment following multi-donor faecal microbiota transplantation in two randomised clinical trials. Gut Microbes. 2025;17:2597628. doi: 10.1080/19490976.2025.2597628. 41378445 PMC12710892

[13] McCallum G , Tropini C . The gut microbiota and its biogeography. Nat Rev Microbiol. 2024;22:105–118. doi: 10.1038/s41579-023-00969-0. 37740073

[14] Donaldson GP , Lee SM , Mazmanian SK . Gut biogeography of the bacterial microbiota. Nat Rev Microbiol. 2015;14:20–32. doi: 10.1038/nrmicro3552. 26499895 PMC4837114

[15] Pereira FC , Berry D . Microbial nutrient niches in the gut. Environ Microbiol. 2017;19:1366–1378. doi: 10.1111/1462-2920.13659. 28035742 PMC5412925

[16] Shepherd ES , DeLoache WC , Pruss KM , Whitaker WR , Sonnenburg JL . An exclusive metabolic niche enables strain engraftment in the gut microbiota. Nature. 2018;557:434–438. doi: 10.1038/s41586-018-0092-4. 29743671 PMC6126907

[17] Moyne O , Norton GJ , Al-Bassam M , Lieng C , Thiruppathy D , Kumar M , Haddad E , Weng Y , Raffatellu M , Zaramela LS , et al. Predicting competition and substrate preferences for targeted microbiome alteration. Cell. 2026;189:3324–3338.e23. doi: 10.1016/j.cell.2026.03.036. 41999742 PMC13105274

[18] Régimbeau A , Budinich M , Larhlimi A , Pierella Karlusich JJ , Aumont O , Memery L , Bowler C , Eveillard D , Jordan F . Contribution of genome‐scale metabolic modelling to niche theory. Ecol Lett. 2022;25:1352–1364. doi: 10.1111/ele.13954. 35384214 PMC9324083

[19] Diener C , Gibbons SM , Resendis-Antonio O . MICOM: metagenome-scale modeling to infer metabolic interactions in the gut microbiota. mSystems. 2020;5: doi: 10.1128/msystems.00606-19. PMC6977071 31964767

[20] Molina Ortiz JP , McClure DD , Holmes A , Rice SA , Read MN , Shanahan ER . Genome-scale metabolic modelling of human gut microbes to inform rational community design. Gut Microbes. 2025;17:2534673. doi: 10.1080/19490976.2025.2534673. 40685566 PMC12283000

[21] Quinn-Bohmann N , Wilmanski T , Sarmiento KR , Levy L , Lampe JW , Gurry T , Rappaport N , Ostrem EM , Venturelli OS , Diener C , et al. Microbial community-scale metabolic modelling predicts personalized short-chain fatty acid production profiles in the human gut. Nat Microbiol. 2024;9:1700–1712. doi: 10.1038/s41564-024-01728-4. 38914826 PMC11841136

[22] Carr AV , Baliga NS , Diener C , Gibbons SM . Personalized clostridioides difficile colonization risk prediction and probiotic therapy assessment in the human gut. Cell Syst. 2025;16:101367. doi: 10.1016/j.cels.2025.101367. 40774255 PMC12497417

[23] Urtecho G , Moody T , Huang Y , Sheth RU , Richardson M , Descamps HC , Kaufman A , Lekan O , Zhang Z , Velez-Cortes F , et al. Spatiotemporal dynamics during niche remodeling by super-colonizing microbiota in the mammalian gut. Cell Syst. 2024;15:1002–1017.e4. doi: 10.1016/j.cels.2024.10.007. 39541983 PMC12066173

[24] Machado D , Andrejev S , Tramontano M , Patil KR . Fast automated reconstruction of genome-scale metabolic models for microbial species and communities. Nucleic Acids Res. 2018;46:7542–7553. doi: 10.1093/nar/gky537. 30192979 PMC6125623

[25] Quinn-Bohmann N , Carr A.V. , Diener C , Gibbons Sean . Moving from genome-scale to community-scale metabolic models for the human gut microbiome. Nature Microbiology. 2025;10. doi: 10.1038/s41564-025-01972-2. PMC13015517 40217129

[26] Plata G , Henry CS , Vitkup D . Long-term phenotypic evolution of bacteria. Nature. 2014;517:369–372. doi: 10.1038/nature13827. 25363780

[27] Eren AM , Esen ÖC , Quince C , Vineis JH , Morrison HG , Sogin ML , Delmont TO . Anvi’o: an advanced analysis and visualization platform for 'omics data. PeerJ. 2015;3:e1319. doi: 10.7717/peerj.1319. 26500826 PMC4614810

[28] Eren AM , Kiefl E , Shaiber A , Veseli I , Miller SE , Schechter MS , Fink I , Pan JN , Yousef M , Fogarty EC , et al. Community-led, integrated, reproducible multi-omics with anvi’o. Nat Microbiol. 2021;6:3–6. doi: 10.1038/s41564-020-00834-3. 33349678 PMC8116326

[29] Sperber AD , Bangdiwala SI , Drossman DA , Ghoshal UC , Simren M , Tack J , Whitehead WE , Dumitrascu DL , Fang X , Fukudo S , et al. Worldwide prevalence and burden of functional gastrointestinal disorders, results of Rome foundation global study. Gastroenterology. 2021;160:99–114.e3. doi: 10.1053/j.gastro.2020.04.014. 32294476

[30] Sun Q , Jia Q , Song L , Duan L . Alterations in fecal short-chain fatty acids in patients with irritable bowel syndrome: a systematic review and meta-analysis. Medicine (Baltimore). 2019;98:e14513. doi: 10.1097/MD.0000000000014513. 30762787 PMC6408019

[31] Villanueva-Millan MJ , Leite G , Wang J , Morales W , Parodi G , Pimentel ML , Barlow GM , Mathur R , Rezaie A , Sanchez M , et al. Methanogens and hydrogen sulfide producing bacteria guide distinct gut microbe profiles and irritable bowel syndrome subtypes. Am J Gastroenterol. 2022;117:2055–2066. doi: 10.14309/ajg.0000000000001997. 36114762 PMC9722381

[32] Aumpan N , Watanabe J , Yuan Y , Kanno T , Leontiadis GI , Chan FK , Moayyedi P . Fecal microbiota transplantation for symptom improvement in patients with irritable bowel syndrome: systematic review and meta-analysis of randomized controlled trials. Gastroenterology. 2026. doi: 10.1053/j.gastro.2026.04.039. 42162772

[33] El-Salhy M , Hatlebakk JG , Gilja OH , Bråthen Kristoffersen A , Hausken T . Efficacy of faecal microbiota transplantation for patients with irritable bowel syndrome in a randomised, double-blind, placebo-controlled study. Gut. 2020;69:859–867. doi: 10.1136/gutjnl-2019-319630. 31852769 PMC7229896

[34] Aroniadis OC , Brandt LJ , Oneto C , Feuerstadt P , Sherman A , Wolkoff AW , Kassam Z , Sadovsky RG , Elliott RJ , Budree S , et al. Faecal microbiota transplantation for diarrhoea-predominant irritable bowel syndrome: a double-blind, randomised, placebo-controlled trial. Lancet Gastroenterol Hepatol. 2019;4:675–685. doi: 10.1016/S2468-1253(19)30198-0. 31326345

[35] Goll R , Johnsen PH , Hjerde E , Diab J , Valle PC , Hilpusch F , Cavanagh JP . Effects of fecal microbiota transplantation in subjects with irritable bowel syndrome are mirrored by changes in gut microbiome. Gut Microbes. 2020;12:1794263. doi: 10.1080/19490976.2020.1794263. 32991818 PMC7583512

[36] Johnsen PH , Hilpüsch F , Cavanagh JP , Leikanger IS , Kolstad C , Valle PC , Goll R . Faecal microbiota transplantation versus placebo for moderate-to-severe irritable bowel syndrome: a double-blind, randomised, placebo-controlled, parallel-group, single-centre trial. Lancet Gastroenterol Hepatol. 2018;3:17–24. doi: 10.1016/S2468-1253(17)30338-2. 29100842

[37] Magnúsdóttir S , Heinken A , Kutt L , Ravcheev DA , Bauer E , Noronha A , Greenhalgh K , Jäger C , Baginska J , Wilmes P , et al. Generation of genome-scale metabolic reconstructions for 773 members of the human gut microbiota. Nature Biotechnology. 2016;35:81–89. doi: 10.1038/nbt.3703. 27893703

[38] Wilson BC , Vatanen T , Cutfield WS , O’Sullivan JM . The super-donor phenomenon in fecal microbiota transplantation. Front Cell Infect Microbiol. 2019;9:2. doi: 10.3389/fcimb.2019.00002. 30719428 PMC6348388

[39] Corral López R , Bonachela JA , Dominguez-Bello MG , Manhart M , Levin SA , Blaser MJ , Muñoz MA . Imbalance in gut microbial interactions as a marker of health and disease. Science. 2026;391:890–895. doi: 10.1126/science.ady1729. 41747050

[40] Li SS , Zhu A , Benes V , Costea PI , Hercog R , Hildebrand F , Huerta-Cepas J , Nieuwdorp M , Salojärvi J , Voigt AY , et al. Durable coexistence of donor and recipient strains after fecal microbiota transplantation. Science. 2016;352:586–589. doi: 10.1126/science.aad8852. 27126044

[41] Rivera-Chávez F , Zhang LF , Faber F , Lopez CA , Byndloss MX , Olsan EE , Xu G , Velazquez EM , Lebrilla CB , Winter SE , et al. Depletion of butyrate-producing clostridia from the gut microbiota drives an aerobic luminal expansion of salmonella. Cell Host Microbe. 2016;19:443–454. doi: 10.1016/j.chom.2016.03.004. 27078066 PMC4832419

[42] Litvak Y , Byndloss MX , Bäumler AJ . Colonocyte metabolism shapes the gut microbiota. Science. 2018;362, 10.1126/science.aat9076. PMC6296223 30498100

[43] Aggarwala V , Mogno I , Li Z , Yang C , Britton GJ , Chen-Liaw A , Mitcham J , Bongers G , Gevers D , Clemente JC , et al. Precise quantification of bacterial strains after fecal microbiota transplantation delineates long-term engraftment and explains outcomes. Nat Microbiol. 2021;6:1309–1318. doi: 10.1038/s41564-021-00966-0. 34580445 PMC8993687

[44] Poyet M , Groussin M , Gibbons SM , Avila-Pacheco J , Jiang X , Kearney SM , Perrotta AR , Berdy B , Zhao S , Lieberman TD , et al. A library of human gut bacterial isolates paired with longitudinal multiomics data enables mechanistic microbiome research. Nat Med. 2019;25:1442–1452. doi: 10.1038/s41591-019-0559-3. 31477907

[45] Chen S , Zhou Y , Chen Y , Gu J . Fastp: an ultra-fast all-in-one FASTQ preprocessor. Bioinformatics. 2018;34:i884–i890. doi: 10.1093/bioinformatics/bty560. 30423086 PMC6129281

[46] Wood DE , Lu J , Langmead B . Improved metagenomic analysis with kraken 2. Genome Biol. 2019;20:257. doi: 10.1186/s13059-019-1891-0. 31779668 PMC6883579

[47] Li H . Minimap2: pairwise alignment for nucleotide sequences. Bioinformatics. 2018;34:3094–3100. doi: 10.1093/bioinformatics/bty191. 29750242 PMC6137996

[48] Love MI , Huber W , Anders S . Moderated estimation of fold change and dispersion for RNA-seq data with DESeq2. Genome Biol. 2014;15:550. doi: 10.1186/s13059-014-0550-8. 25516281 PMC4302049

